# Patient experiences of internet-based enhanced cognitive behavior therapy for eating disorders

**DOI:** 10.1016/j.invent.2025.100801

**Published:** 2025-01-23

**Authors:** Anne-Charlotte Wiberg, Ata Ghaderi, Thomas Parling, Magdalena Jansson, Elisabeth Welch

**Affiliations:** a Centre for Psychiatry Research, Department of Clinical Neuroscience, Karolinska Institutet, Stockholm Health Care Services, Stockholm County Council, Stockholm, Norra stationsgatan 69, 113 64 Stockholm, Sweden; b Division of Psychology, Department of Clinical Neuroscience, Karolinska Institutet, Nobels väg 9, 17177 Stockholm, Sweden; c Stockholm Center for Eating Disorders, Stockholm County Council, Wollmar Yxkullsgatan 27B, 118 50 Stockholm, Sweden; d Global Health and Migration Unit, Department of Women's and Children's Health, Uppsala University, Akademiska sjukhuset, 751 85 Uppsala, Sweden

**Keywords:** Patient experiences, Internet-delivered therapy, Enhanced cognitive behavioral therapy, Binge-eating, Eating disorders, Qualitative research

## Abstract

**Background:**

Internet-based Cognitive Behavioral Therapy (ICBT) has shown promise in addressing the treatment gap for eating disorders (EDs), with evidence indicating moderate to large effect sizes. However, some individuals experience no improvement or deterioration in their condition, highlighting the need to understand both successful and unsuccessful outcomes.

**Aim:**

This study aimed to explore patients' experiences undergoing Internet-based guided self-help treatment based on Enhanced Cognitive Behavior Therapy (ICBT-E) for bulimia nervosa (BN) and binge eating disorder (BED), focusing on both those who benefited from the treatment and those who did not.

**Method:**

Sixteen participants with a diagnosis of full or subthreshold BN or BED, including eight non-responders and eight responders, participated in a semi-structured telephone interview. Data were analyzed using qualitative content analysis.

**Results:**

Responders strongly identified with the content, facilitating treatment implementation, while non-responders found the content less relevant to their symptoms. The treatment was overall perceived as time-consuming, but non-responders found it overwhelming and struggled with motivation and self-discipline. Non-responders preferred synchronous communication, while responders valued the flexibility of asynchronous contact. Overall, participants acknowledged the importance of ICBT-E, though non-responders felt it was not tailored to their specific needs.

**Conclusions:**

The study highlighted considerations for designing and implementing ICBT-E, including tailoring content to diverse patient symptoms, managing time demands, and considering motivation and self-discipline when assigning this treatment. While ICBT-E shows promise for the widespread dissemination of treatment for EDs, ongoing evaluation of progress during treatment and timely referral to alternative interventions for non-responders are crucial for optimizing outcomes.

## Highlights

• Patients with eating disorders participated in a qualitative interview after ICBT-E treatment.
• The treatment was seen as time-consuming, with non-responders finding it overwhelming
• Non-responders faced greater difficulties with motivation and self-discipline.
• Responders valued flexible asynchronous contact and non-responders real-time dialogue.
• Non-responders felt that ICBT-E wasn't sufficiently tailored to their specific needs.

## 1 Introduction

Internet-based Cognitive Behavioral Therapy (ICBT) treatment holds the promise of bridging the gap between individuals in need of psychiatric care and the availability of evidence-based treatments (Andersson et al., 2019). In the context of eating disorders (EDs), one of the most serious psychiatric disorders, this advancement can contribute to addressing the challenge where around 80 % of affected individuals lack access to treatment (Dobrescu et al., 2020; Hart et al., 2011). The evidence base supporting ICBT for psychiatric disorders is well established. An updated meta-analysis of 31 trials, including bulimia nervosa (BN) and binge eating disorders (BED), found outcomes comparable to face-to-face CBT (Hedman-Lagerlöf et al., 2023). ICBT for BN and BED significantly reduce core clinical symptoms, including dietary restraint and concerns about shape, eating, and weight, as well as the frequency of binge eating episodes (Zhong et al., 2024). ICBT is generally perceived as an acceptable treatment option for individuals with EDs (Agras et al., 2017; McClay et al., 2016). However, while the majority of patients' experience positive outcomes from ICBT, some individuals experience absence of effect from the treatment and some report a deterioration in their condition. Research indicates that approximately 6 % of individuals undergoing ICBT for psychiatric conditions, including anxiety disorders and depression, experience deterioration (Rozental et al., 2017), while around 27 % show no response to treatment (Rozental et al., 2019). Therefore, understanding the perspectives of both successful and unsuccessful cases in ICBT may help inform future intervention development and provide knowledge that facilitates allocation to appropriate treatments.

Qualitative research provides a valuable approach to explore patients' experiences, uncovering factors that influence treatment outcomes, including both facilitators and barriers. A study on CBT-T, a brief form of CBT for non-underweight patients with EDs delivered face-to-face (Waller et al., 2018), highlighted patients' positive experiences with its caring yet firm and fair therapeutic relationship, adaptability to individual needs, and benefits for eating behaviors and quality of life. Patients with prior experience of treatment viewed CBT-T positively. While weekly sessions supported engagement, challenges included balancing therapy with daily responsibilities and experiencing the length of the CBT-T as too short (Hoskins et al., 2019).

Expanding the focus to ICBT for EDs, several qualitative studies have shed light on patients' experiences. Research on the program Overcoming Bulimia Online (Williams et al., 1998) highlighted participants' appreciation of the program's anonymity and flexibility but also noted the need for self-discipline and motivation. Email support was essential for maintaining engagement, and continued therapist contact post-intervention was recommended (McClay et al., 2013; Sánchez-Ortiz et al., 2011). Challenges with the program included technical issues, high workload demands, insufficient tailoring to participants' individual needs (McClay et al., 2013), and the need for earlier intervention and more varied examples to better support recovery (Sánchez-Ortiz et al., 2011).

Further research on ICBT for EDs has examined the Student Bodies program (Jacobi et al., 2012), identifying critical factors influencing usability and engagement. Usability was highlighted through three main themes: layout, navigation, and content, while engagement was addressed through support and engagement conditions. The study demonstrated that testing and refining the intervention design can improve both usability and engagement (Nitsch et al., 2016).

Building on this foundation, the everyBody program, a more recent version of Student Bodies has been investigated. One study focused on everyBody Plus, an adapted version targeting adult women with BN, BED, and other specified feeding and eating disorders characterized by binge eating. Participants described the program as easy to integrate into daily life and reported positive effects on cognitive, emotional, interpersonal, and behavioral aspects. However, they also noted that its suitability varied depending on individual demographic and clinical characteristics, suggesting a need for tailored approaches in future interventions (Yim et al., 2020).

Finally, a study investigated the everyBody for students in Aotearoa New Zealand with current or past experiences of binge eating and/or compensatory purging behaviors. The program was found to be both acceptable and feasible, though participants emphasized the need for cultural adaptations and greater flexibility to address individual needs (Mitlash et al., 2024).

Notably, several of the qualitative studies evaluating ICBT for EDs include participants who are either intervention completers or individuals with positive treatment outcomes, which do not represent the broader population of intervention users (Yim et al., 2020; Yim and Schmidt, 2019). Research on the experiences of individuals who have not benefited from ICBT for EDs is limited. Recent studies emphasize the importance of comparisons between improved and non-improved psychotherapy patients (Werbart et al., 2015), highlighting the need for further research to better address the diverse needs and experiences in ICBT for EDs.

The present study aimed to examine the experiences of patients diagnosed with either full or subthreshold BN or BED who underwent internet-delivered guided self-help. The treatment was based on Enhanced Cognitive Behavioral Therapy (CBT-E), an evidence-based approach for EDs (Fairburn, 2008). In a single-group open trial conducted by Wiberg et al. (2022), the treatment demonstrated a completion rate of 73.2 %. At the 3-month follow-up, 55.6 % of participants had achieved recovery, 8.3 % were classified as non-reliably recovered, 8.3 % showed improvement, and 27.8 % remained unchanged (Wiberg et al., 2022).

Specifically, this study aimed to investigate the experiences of patients who benefited from internet-delivered CBT-E (ICBT-E) for EDs, as well as those who did not respond to the treatment.

## 2 Method

A qualitative design employing semi-structured interviews was chosen to address the aim of the study. The interviews were analyzed using inductive content analysis. The study was reported following the COREQ (Consolidated Criteria for Reporting Qualitative Research) guidelines (Tong et al., 2007). Ethical approval for the study was obtained from the Swedish Ethical Review Authority (2022–02859-02).

### 2.1 Participants and setting

Participants were adults seeking care at the Stockholm Center for Eating Disorders (SCED) in Stockholm County, Sweden. They were assessed according to clinic routines by well-trained clinicians using the Mini International Neuropsychiatric Interview (MINI; Sheehan et al., 1998). Patients meeting criteria for full or subthreshold BN or BED according to DSM-5 (American Psychiatric Association, 2013) were invited via telephone by one of the authors (AW) to participate in the study. Purposive sampling was employed to recruit participants exhibiting both significant symptomatic change and those with little or no change. Notably, the participants in the current study were distinct from those involved in the study by Wiberg et al. (Wiberg et al., 2022).

Responders and non-responders were identified by facilitators through progress-related information derived from the treatment. Treatment response was defined using the Eating Disorders Examination Questionnaire (EDE-Q), which measures key behavioral features and core psychopathology of EDs across four subscales and a global score (Fairburn and Beglin, 2008). Additionally, global improvement assessed by experienced clinicians using the Clinical Global Impression-Improvement scale (CGI—I; Guy, 1976) was utilized due to missing EDE-Q data in some cases, particularly among non-responders. For patients with post-treatment data, outcomes were evaluated using the Reliable Change Index (Ekeroth and Birgegård, 2014; Wise, 2004) and clinical cut-off based on Swedish norms for the EDE-Q (Welch et al., 2011). The RCI calculation, based on a test-retest reliability of 0.83, provided a standard error of 0.52 and a standard deviation of 0.74, determining meaningful change. A clinical cut-off score of 2.76 on the global EDE-Q was used. Remission was defined as a reliable change (RCI Z ≥ 1.96) paired with a final score below the cut-off. Patients with RCI < 1.28 were classified as not improved, while those with RCI ≥ 1.96 in the negative direction were classified as deteriorated (Ekeroth and Birgegård, 2014). For patients with missing post-treatment data, those rated as much improved or very much improved on the CGI—I were categorized as treatment responders. Conversely, patients classified as showing no change, minimally worse, much worse, or very much worse on the CGI-I were considered non-responders.

Out of the 32 patients who were contacted, 24 were successfully reached. Among them, 23 expressed their willingness to participate in the study, while one declined without providing a reason. Additionally, six patients dropped out before providing informed consent and could not be reached after the initial contact. Subsequently, 17 patients provided informed consent to participate in the study. However, one patient could not be reached, leaving 16 patients - eight responders and eight non-responders - who were successfully contacted by telephone and participated in the interviews.

The study was conducted at SCED and the research team consisted of three women and two men: a licensed psychotherapist and PhD-student working with ED treatment at SCED (AW), a psychologist working with ED treatment at SCED (MJ), and three professionals who are both psychologists and researchers (EW, Associate Professor, AG, Professor and TP, PhD) with extensive experience of working with and conducting research on EDs.

### 2.2 The treatment

Treatment interventions included psychoeducation, personal formulation of the processes maintaining the ED, establishment of healthy eating patterns, and targeted over-evaluation of shape and weight, as well as dietary restraint (Fairburn, 2008). ICBT-E lasted for 12–15 weeks, with weekly facilitator feedback providing guidance and support for patient progress. During the midpoint of the treatment, an assessment of the patient's progress and level of engagement with the treatment was conducted. If insufficient progress was observed, the possibility of discontinuing the treatment or transitioning to alternative treatment options was considered. At the end of treatment, participants had access to the treatment content for six months, and a video follow-up session was conducted three months post-treatment. For a more detailed description of the treatment components, see Wiberg et al. (2022).

### 2.3 Procedure

Written and verbal information about the study was provided to the participants after completing ICBT-E, allowing them the opportunity to ask questions before giving informed consent. Participants that consented to participate were then asked to complete a socio-demographic questionnaire providing information on age, computer literacy, education, employment status, income and area of residence. Following this, semi-structured interviews were conducted via telephone by four research assistants who were external to both the research team and SCED's staff, fostering an environment where participants would feel comfortable to openly express their treatment experiences and experience of interactions with facilitators. The interview guide covered areas such as participants' overall experiences with ICBT-E, their perceptions of facilitator support, helpful and less helpful aspects of the treatment, and suggestions for improvement. Participants were also asked about any negative effects they attributed to the treatment and their thoughts on undergoing treatment via the internet. A full interview guide is provided in the online supplement. The interviews lasted approximately 30–40 min and were conducted between May and September 2023.

### 2.4 Analysis

The interviews were audio recorded and transcribed verbatim by AW. Qualitative content analysis (Graneheim et al., 2017; Graneheim and Lundman, 2004) with an inductive approach was used focusing on the manifest content in the interviews. Qualitative content analysis serves as a valuable method for describing phenomena that have not been previously studied or when existing research and knowledge are limited. As the initial step, AW thoroughly read and re-read the interviews to gain an overall understanding of the text. Based on the purpose of the study, meaning units were identified which were then condensed and labeled with codes by AW (see Table 1).

**Table 1:** Example of the analytical process.

Main category	Subcategory	Sub-subcategory	Code	Meaning units
Treatment modality	Communication	Personal or general	Unexpectedly personal	“I thought it would be a bit more standard, but the feedback was very personal, and I have appreciated that very much.” (5R)

Notes: R = responder.

To enhance the credibility of the analysis, regular meetings between AW, MJ and EW were held throughout the analysis process. Additionally, three transcripts were double coded by MJ and AW, followed by comparison and discussion of their individual coding choices. Refinements were made based on these discussions, and AW abstracted the codes into preliminary main categories, subcategories and sub-subcategories. Subsequent discussions with all authors, both individually and collaboratively, led to further adjustments. The final main categories, subcategories and sub-subcategories were collaboratively developed with the research team, ensuring a comprehensive representation of the entire dataset in the findings and enhancing the credibility of the study. In order to explore potential differences in content aspects between responders and non-responders, the analysis was initially conducted on the complete dataset before being separately undertaken for each group.

Microsoft Excel and NVivo 12 software package, produced by QSR International, was used for coding and analysis.

## 3 Results

### 3.1 Participant demographics and characteristics

Saturation in the data was achieved during the 16 interviews, meaning, no substantial new information emerged during the two last interviews. The characteristics of the patients are presented in Table 2.

**Table 2:** The characteristics of the participants.

Participants	Total sample (N = 16)	Non-responders (N = 8)	Responders (N = 8)
Age *M* (*SD*; range)	37.25 (10.5; 23–57)	34.38 (10.0; 23–57)	40.13 (10.9; 25–56)


Participants	Total sample (N = 16)	Non-responders (N = 8)	Responders (N = 8)
	N (%)	N (%)	N (%)
Gender			
Female	13 (81.2)	6 (75.0)	7 (87.5)
Male	3 (18.8)	2 (25.0)	1 (12.5)
Education level			
Nine-year compulsory school	2 (12.5)	1 (12.5)	1 (12.5)
High school	3 (18.8)	1 (12.5)	2 (25.0)
University and above	11 (68.7)	6 (75.0)	5 (62.5)
Occupation			
Student	1 (6.3)	0	1 (12.5)
Employed	12 (75.0)	7 (87.5)	5 (62.5)
Long-term sick leave	2 (12.5)	1 (12.5)	1 (12.5)
Unemployed	1 (6.3)	0	1 (12.5)
Diagnosis			
BN	7 (43.7)	4 (50.0)	3 (37.5)
BED	6 (37.5)	3 (37.5)	3 (37.5)
Subthreshold BN or BED	3 (18.8)	1 (12.5)	2 (25.0)
BMI *M* (*SD*; range)	33.85 (7.30; 20.20–49.70)	32.12 (7.58; 20.20–46.60)	35.49 (7.12; 28.40–49.70)

Note: BN = bulimia nervosa, BED = binge eating disorder.

### 3.2 Main categories, subcategories and sub-subcategories

The content analysis generated three main categories, six subcategories and 14 sub-subcategories (see Table 3). The three main categories were “Enhancing and impeding content”, “Contextual factors” and “Treatment modality”.

**Table 3:** Overview of main categories, subcategories and sub-subcategories, with the number of participants represented in each sub-subcategory.

Main categories	Subcategories	Sub-subcategories	Total sample (N = 16)	Non-responders (N = 8)	Responders (N = 8)
Enhancing and impeding content	Effectiveness of intervention strategies	Impact of expert involvement	4	3	1
		Core CBT-E interventions	16	8	8
	Relatability to treatment content	Personally addressed	8	1	7
		Insufficient relevance	7	5	2
Contextual factors	External contextual factors	Technology and design	12	6	6
		Environments	4	2	2
		Aspects of time	7	3	4
	Internal contextual factors	Fear of failure	6	5	1
		Motivation and self-discipline	13	6	7
		Psychiatric comorbidity	7	4	3
Treatment modality	Communication	Personal or general	15	7	8
		Real time or delayed	14	8	6
	Internet-based	Good but not suitable for everyone	16	8	8
		Flexibility and availability	13	7	6

Within “Enhancing and impeding content”, the subcategory “Effectiveness of intervention strategies” emerged, with the sub-subcategories “Impact of expert involvement” and “Core CBT-E interventions”. Additionally, the subcategory “Relatability to treatment content” included the sub-subcategories “Personally addressed” and “Insufficient relevance”.

Contextual factors” encompassed two subcategories, each comprising three sub-subcategories. “External contextual factors” included “Technology and design”, “Environments”, and “Aspects of time” while “Internal contextual factors” comprised “Fear of failure”, “Motivation and self-discipline”, and “Comorbidity”.

Lastly, the main category “Treatment modality” included the subcategory “Communication”, with the sub-subcategories “Personal or general” and “Real-time or delayed”, as well as the subcategory “Internet-based”, with sub-subcategories “Good but not suitable for everyone” and “Flexibility and availability”.

Both responders and non-responders provided descriptions of their experiences that could be categorized into the same categories. However, it is notable that within some categories, divergent perspectives emerged from the two groups containing a dichotomy in the same aspect. Quotes from participants that illustrate the main categories, subcategories, and sub-subcategories are presented with the participant's number and whether they are non-responders (NR) or responders (R).

### 3.3 Enhancing and impeding content (main category)

The category of “Enhancing and impeding content” encompassed experiences of treatment elements such as exercises, psychoeducation and videos of role playing, that either facilitated or hindered engagement and the process of change within the treatment.

#### 3.3.1 Effectiveness of intervention strategies (subcategory)

Many participants found the psychoeducation provided in the treatment to be a powerful intervention, along with the video-recorded role-plays with patients and a therapist. Among the views expressed was that the participation of researchers and clinicians gave greater confidence in the treatment and the role-plays were perceived as authentic. Furthermore, the rationale for change were described as informative, clear, and conducive to improvements, such as establishing regular eating habits. Other interventions that both non-responders and responders reported as facilitating behavioral changes included psychoeducation about the physiological consequences of EDs, regular eating, and engagement in alternative activities.

Self-monitoring of meals was highlighted as a facilitator, but only by responders. They stated that the self-monitoring contributed to increased awareness of eating patterns requiring change. On the contrary, four non-responders found self-monitoring to be a barrier in treatment. They expressed that it was challenging, demanding, and resulted in heightened stress. Moreover, it increased their preoccupation with food, which they perceived to result in deterioration. One non-responder reported that self-monitoring of binge eating episodes triggered impulses to compensate.

“*And I think it was difficult to write down the binge eating as well, then it was really strange. ‘Yes, but you have eaten too much of this’ and in my head, what is it like, ‘well now I am messing up, how can I get rid of this in the best way?’*”

(16NR)

Among the interventions considered challenging, weekly weighing at home was also mentioned, with participants expressing difficulties arising from distress associated with confronting their weight, particularly among those at a higher weight.

Several participants at a higher weight expressed a desire to lose weight to reduce associated health risks. Some expressed that weekly confrontations with their weight served as a painful reminder that despite adopting healthier eating habits, their weight remained unchanged. Despite the challenges posed by this intervention, it was noted as necessary and beneficial for reducing weight-controlling behaviors or avoidance.

One participant expressed dissatisfaction with the intervention aimed at addressing “Feeling fat”, perceiving it as ill-suited for individuals at a higher weight. It was perceived that the exercise implied acceptance of higher weight, notwithstanding the plans to seek weight reduction assistance after completing ICBT-E. This exercise generated significant distress over an extended period, prompting the participant to consider discontinuing the treatment.

#### 3.3.2 Relatability to treatment content (subcategory)

An important distinction between responders and non-responders was their ability to relate to the content regarding the symptoms addressed. Responders often identified strongly with the content, especially in role-plays, which heightened their awareness of ED behaviors and facilitated the implementation of interventions.

“*When I tried to come up with excuses, they were sort of included in these short films. It was as if I was sitting in that armchair together with the therapist who was sitting with another client in the film.*”

(2R)

Conversely, feedback from non-responders highlighted a scarcity of material addressing their specific symptoms, such as compensation through fasting or concerns related to high weight. They also highlighted an excessive emphasis on symptoms that were perceived as irrelevant and unhelpful, such as compensatory behaviors, for individuals with BED.

“*I didn't really feel that all the information was directed at me and my issues, so it wasn't always that I felt this was relevant to me and almost made me feel that it didn't really help me […] In some way, that you maybe didn't belong there or that it wasn't the right program*”

(11NR)

One participant felt that the treatment was overly focused on self-induced vomiting as a compensatory strategy, which she reported had led to an increase in the frequency of this behavior. The participant, along with others, expressed a need for more tailored content addressing their specific problems.

### 3.4 Contextual factors (main category)

Contextual factors encompassed the external and internal conditions and circumstances that surrounded participants, influencing their treatment experience.

#### 3.4.1 External contextual factors (subcategory)

In terms of technology and design, several participants, including both responders and non-responders, appreciated the program's integration of various media formats, particularly emphasizing the availability of treatment content primarily through videos.

“*For me personally, video is the medium that I find easier to consume because I have difficulty with concentration […] video reaches both your hearing and your sight […] communication becomes easier and it's very good that it's offered complemented with text. It would have been very difficult if it had only been long texts.*”

(9NR)

However, both groups reported technical challenges, such as difficulties using the treatment via smartphones and logging in with electronic identification for real-time daily monitoring. While recognizing the necessity of robust security measures like electronic identification for safeguarding healthcare data, many participants advocated for a mobile application to enhance adherence to the registration process.

Some participants, both responders and non-responders, noted varying adherence to treatment across different environments. They found adherence more feasible at home than in other settings like workplaces or social gatherings. Moreover, participants reported feelings of discomfort and shame associated with eating when others did not, which further affected their adherence to the regular eating intervention.

Comments indicating that the treatment was time-consuming were provided by both responders and non-responders. Non-responders found the commitment overwhelming, struggling to find time for the included exercises and to balance work and childcare. Some felt burdened by the gradual integration of modules and interventions, experiencing stress due to the accumulation of knowledge and tasks.

“*There were just too many parallel things, it kept building up all the time. At the beginning, it was module 1, but I managed that, and then I'm done with that, and the next week it's module 2, but then in week three you have to do both module 2 and 3, and in week four you have to do 2, 3, and 4 […] it increased exponentially in a way that I didn't really feel I could do justice to.*”

(10NR)

Responders also acknowledged the program's time demands. One noted that being unemployed helped in absorbing the treatment effectively. Despite the time commitment exceeding expectations, responders recognized its necessity for achieving positive outcomes. In contrast to non-responders, responders appreciated the sequential module completion, finding comfort in the step-by-step acquisition of information and in finishing one module before proceeding to the next.

#### 3.4.2 Internal contextual factors (subcategory)

Among internal contextual factors, fear of failure was prominent, particularly among non-responders. They feared feeling like failures if they deviated from their meal plans, worrying it might trigger binge eating episodes. Missing tasks, like recording meals, intensified these feelings, sometimes leading to avoidance of the program. For some, meal planning resembled past disorder-driven behaviors, further triggering feelings of failure.

Both responders and non-responders highlighted motivation and self-discipline as critical factors. Non-responders struggled with maintaining focus, structure, and engagement, often procrastinating, and skipping tasks. In contrast, responders highlighted motivation in terms of determination, a refusal to give up, and a desire to improve as contributing factors to program completion.

Non-responders highlighted how psychiatric comorbidities like ADHD posed challenges in managing program exercises independently, expressing the need for additional support. They also noted anxiety and depression as impending factors, emphasizing the importance of addressing these conditions simultaneously. Similarly, responders highlighted the need to address psychiatric comorbidities concurrently with ICBT-E.

“*One thing that helped me was that while I was waiting to get the treatment, I got help from the health center with medication for my depression and anxiety […] because I think if I hadn't received help with medication for depression and anxiety before the treatment, it wouldn't have gone as well as it did. Because if you're really depressed, you probably can't benefit from the treatment.*”

(5R)

### 3.5 Treatment modality (main category)

The main category “Treatment Modality” encompassed participants' experience of the digital delivery of treatment.

#### 3.5.1 Communication (subcategory)

Many participants, both non-responders and responders, were highly satisfied with facilitators' support. They valued their accessibility, expertise, thorough engagement in exercises and messages, and felt genuinely understood despite never having met in person. They appreciated the caring nature and balanced encouragement provided by facilitators, whose personalized approach exceeded expectations. On the contrary, some non-responders did not perceive as much benefit from the facilitator's support, instead finding more value in the program's content.

“*Well, I think the therapist wrote well […] but, entirely, it hasn't really sunk in […] now that I think about it, I don't feel anything about it. The therapist was really good and all, but no, I don't feel anything there. I probably got more out of the videos and the reading.*”

(15NR)

They wanted more encouraging feedback on their treatment progress. Some felt the facilitator's contact was impersonal and their feedback too generalized, requesting more personalized comments tailored to their experiences and needs.

Non-responders preferred face-to-face meetings with facilitators, either alone or combined with messaging. They found weekly asynchronous messaging insufficient and desired more interactive dialogue for back-and-forth discussions. Verbal communication was viewed as more effective than written, as they struggled to express themselves through writing.

A couple of responders desired video or phone contact, one for better support in difficult situations and another for the opportunity to be honest about feelings and receive focused help during treatment. On the other hand, several responders expressed appreciation for communication via messages, as the text-based communication resembled a conversation. They perceived comments from the facilitator as contributing to a positive dialogue.

“*The communication we had was always helpful, so it was very positive. When I was waiting for her, you'd get that little ping on the phone, like, ‘You've got a message […]’ and I'd be like, ‘Oh, how nice!’ So it absolutely felt like I had her support, even though it was in a fixed format, you know.*”

(4R)

Some appreciated the convenience of internet communication, with the ability to write to their facilitator at any time without waiting for a scheduled meeting.

#### 3.5.2 Internet-based (subcategory)

Overall, the participants appreciated internet-based treatment modalities, regardless of their response to the treatment. Both responders and non-responders valued the availability of such treatments, though non-responders indicated the format didn't meet their personal needs. Responders were notably surprised by the effectiveness of the treatment in alleviating symptoms.

“*It was even better than I had expected […] because I thought I would get some help along the way and then continue working on it similarly, maybe for a few more years. But now, what is it, ten weeks later, I feel […]well, not free from the eating disorder, but right now, I have no issues.*”

(5R)

Challenges highlighted include feelings of isolation and a desire for more interactive support through a moderated forum. Additionally, there was feedback on the need for addressing how past life experiences had influenced the development of EDs.

Both responders and non-responders, emphasized the convenience of the internet-based treatment modality. They appreciated the flexibility of undergoing treatment at their convenience, without taking time off from work or travel to treatment centers.

*I like internet-based […] for me, it was important. Also, because I live out in the countryside, so maintaining the routine of traveling in once a week or something like that, it just doesn't work […] in the situation I'm in now, so it was very important for me, and it feels really, really good.*

(4R)

This format expedited the initiation of treatment and broadened its reach. Additionally, the anonymity afforded by the online format was highly valued, especially by rural participants. They also found the ability to revisit and review treatment materials highly beneficial for comprehension, an advantage over face-to-face sessions where psychoeducation is usually delivered verbally.

## 4 Discussion

The present study examined the experiences of patients receiving ICBT-E, encompassing individuals who benefited from the program and those who did not. The analysis highlights notable differences between responders and non-responders. Responders found the program beneficial in general while non-responders voiced multiple concerns in all three main categories “Enhancing and impeding content”, “Contextual factors” and “Treatment modality”. The study provides insights into factors influencing engagement and outcomes that may enhance optimal use of ICBT-E in clinical care.

### 4.1 Enhancing and impeding content

The main category Enhancing and impeding content highlighted participants' views on the effectiveness of intervention strategies and the relatability of treatment content. There was a shared perspective on several treatment components, including psychoeducation, regular eating, and alternative activities, which facilitated behavioral changes. However, while self-monitoring of food intake was beneficial for responders, some non-responders found it distressing, perceiving it as worsening their symptoms.

Real-time self-monitoring of food intake is a core intervention in CBT-E (Fairburn, 2008). While it may initially increase preoccupation with food and cause distress, these effects usually decrease as treatment progresses (Fairburn, 2008; Wilson and Vitousek, 1999). Early discontinuation may hinder reductions in food-related preoccupations and associated concerns. Addressing the distress caused by self-monitoring is crucial, as it significantly reduces binge eating in individuals with BN or BED (Latner and Wilson, 2002), and its perceived usefulness is associated with improvements in ICBT (Carrard et al., 2006). Exploring ways to detect these challenges early in treatment is essential to ensure appropriate guidance and support when needed.

Another notable contrast emerged between responders and non-responders in their ability to relate to the program content related to their symptoms. Responders resonated strongly with the material, facilitating behavior change, while non-responders felt less connected, perceiving parts of the material as unhelpful.

Previous studies on digital interventions emphasize the importance of personal relevance, with participants identifying its absence as a reason for disengagement (Perski et al., 2017). Similarly, individuals with EDs express a preference for more personalized digital treatments (Linardon et al., 2021). These findings underscore the importance of adapting treatment to accommodate diverse patient symptoms, potentially enhancing treatment engagement. As an example, the content on compensatory behaviors can be omitted for those with BED. Another alternative is a more fine-grade modular format in which the core components of the programs are delivered to all, while the participants are given the opportunity to choose among other modules that might not be relevant to all.

### 4.2 Contextual factors

The main category Contextual factors captured the impact of external and internal aspects, such as time management, motivation, and comorbidities, on participants' ability to engage with ICBT-E. While both responders and non-responders described challenges related to balancing treatment with work and family responsibilities, non-responders reported greater difficulties. They often felt overwhelmed by the gradual increase in intervention demands, echoing previous findings that time constraints and perceived burden can hinder engagement with digital programs (Rozental et al., 2015, Rozental et al., 2020). Addressing barriers like time constraints and help patients prioritize tasks in line with CBT-E principles is crucial (Fairburn, 2008).

A further difference between the groups was the influence of fear of failure. This fear was particularly prominent among non-responders, often associated with concerns about deviations from meal plans or missed tasks, occasionally leading to program avoidance. This fear has previously been recognized as a factor influencing non-responders negative attitude toward psychotherapy and potentially hindering their engagement (Carrington et al., 2024). Addressing fears during initial assessments through open discussions and normalizing setbacks may improve engagement.

Motivation and self-discipline were identified as critical factors influencing treatment engagement. Responders exhibited determination, enabling them to manage independent tasks effectively, while non-responders faced significant difficulties in these areas. Motivation is a critical factor influencing engagement and effectiveness in digital interventions (O'Connor et al., 2016; Patel et al., 2020). Higher pre-treatment motivation is associated with improved outcomes (Sansfaçon et al., 2020), underscoring the importance of identifying patients with low motivation early.

Both groups also noted the influence of psychiatric comorbidities, including ADHD, anxiety, and depression. Responders found concurrent treatment beneficial for managing ICBT-E and achieving positive outcomes, while non-responders struggled to complete treatment due to these conditions. Comorbidity, such as depression, is linked to engagement difficulties (Yim et al., 2020) and poorer ICBT outcomes for EDs (Wagner et al., 2016). While ICBT can reduce depressive and anxiety symptoms secondary to EDs (Dölemeyer et al., 2013), assessing and monitoring comorbidities is crucial. Tailored treatments targeting comorbid diagnoses should be considered when psychiatric comorbidity hinder progress in ICBT-E (Fairburn, 2008).

### 4.3 Treatment modality

Participants' experiences of the digital delivery of treatment, highlighted in the main category Treatment modality, encompassed communication and treatment via the Internet. Overall, participants expressed high satisfaction with facilitator support, emphasizing engagement, validation, and personalization. However, some non-responders noted limitations, such as insufficient encouragement, impersonal feedback, and a need for tailored interactions. The importance of personalized support tailored to the participant's situation and health status has been highlighted in earlier research (Patel et al., 2020; Sayar et al., 2023). Consequently, it is crucial to ensure that facilitators develop skills to appear attentive and genuinely interested within the communication framework supported by ICBT, as such validation may enhance patient engagement (Sayar et al., 2023).

Preferences regarding real-time or delayed communication with facilitators also varied between the groups. Responders showed a positive attitude toward written asynchronous communication, finding it flexible and convenient. Previous research also noted that participants found written communication advantageous and were surprised by the high quality of the relationship established through online communication in ICBT (Patel et al., 2020). In contrast, non-responders preferred synchronous communication like face-to-face meetings, video calls, or telephone calls, seeking additional support beyond weekly emails, consistent with prior findings (Hadjistavropoulos et al., 2018; Sánchez-Ortiz et al., 2011; Yim et al., 2020).

Studies on communication methods in ICBT have shown mixed outcomes, with reports of benefits from additional telephone support (Pihlaja et al., 2020), while others found no significant differences between synchronous and asynchronous methods (Lindner et al., 2014). Aardoom et al. (2016) reported that variations in therapist support levels or frequency in digital ED interventions had no significant impact on symptomatic improvement or participant satisfaction with the intervention or their therapist. Research on the impact of communication frequency and methods in digital ED interventions is limited (Barakat and Maguire, 2022). Until more effective support strategies are identified, ensuring accessible support in ICBT-E can help optimize resource allocation for patients requiring more intensive or alternative communication methods, including face-to-face meetings.

Both responders and non-responders generally expressed positive attitudes toward the internet-based intervention. Consistent with findings from other studies, its flexibility and accessibility were frequently highlighted (Linardon et al., 2021; Yim et al., 2020, Yim et al., 2021; Yim and Schmidt, 2019). While non-responders acknowledged the value of these interventions, they felt the programs did not fully address their specific needs. This finding aligns with previous research showing that many individuals with EDs view ICBT positively (McClay et al., 2016), although some prefer face-to-face therapy, and its suitability is perceived to depend on individual characteristics and conditions (Yim et al., 2021).

These findings highlight the complexity of matching treatment modalities to individual patient needs. While flexibility and accessibility are clear advantages of ICBT-E, feedback from non-responders emphasizes the importance of understanding and addressing specific barriers to ensure that patients receive interventions tailored to their unique circumstances and needs.

### 4.4 Strengths and limitations

This study provides valuable guidance for program development and treatment allocation in clinical settings for patients with EDs. Conducting the study within a clinical context ensures findings reflect real-world treatment delivery, and the inclusion of quotes enhances credibility and authenticity. Moreover, the study's credibility and trustworthiness are enhanced by the collaborative analysis process and double coding of transcripts by two authors. The authors' involvement in the development and evaluation of the feasibility and preliminary outcome of ICBT-E was managed by maintaining awareness of this fact, maintaining a reflective distance, and using our perspectives to highlight various aspects of the material.

The interviews were conducted by four research assistants who were independent of both the treatment facility and the research team, thereby creating conditions conducive to open expression of experiences. However, this approach also posed a limitation, as different interviewers might have emphasized varied topics and follow-up questions. The use of the same interview guide for all participants, with no major changes made during data collection, minimized the risk of inconsistency by ensuring that all participants were asked about the same areas, thereby enhancing dependability. Finally, the study could have benefited from greater demographic diversity, such as including more male and less-educated participants, to further enrich the findings.

### 4.5 Clinical implications and future research

This study highlights several important implications for optimizing ICBT-E in the treatment of EDs.

To address engagement issues related to the content in ICBT-E, implementing periodic, concise evaluations of program content may offer a viable solution. Such evaluations could help identify and address barriers related to core interventions that are critical for treatment success, like self-monitoring of food intake.

The difference in how responders and non-responders related to the program content highlights the need to adapt program content to better reflect the diverse presentations of EDs, as including examples of the various compensatory behaviors observed in EDs. Offering optional modules, such as content specifically for patients who exhibit compensatory behaviors while allowing those without these behaviors to bypass this material, could further improve both relevance and engagement. Future research should evaluate the effectiveness of these tailored adaptations compared to generic ICBT content for EDs.

Barriers, such as time demands, underscore the need for mid-treatment evaluations to identify patients needing support in task prioritization or referral to alternative treatments. Open discussions addressing fears, expectations, and normalizing setbacks may help mitigate the impact of fear of failure. Additionally, assessing motivation prior to treatment and addressing low engagement early is crucial. For patients facing significant challenges with motivation and self-discipline, more intensive or tailored interventions may be necessary to support their progress effectively. Furthermore, assessing and monitoring comorbidities is essential to ensure appropriate care, with tailored treatments targeting these conditions when they hinder progress.

To enhance the valued experience of feeling genuinely understood while minimizing perceptions of generic and impersonal feedback from facilitators, additional training for facilitators may enhance their ability to provide contextually appropriate feedback, promoting better engagement and outcomes (Sayar et al., 2023).

The mode and frequency of support also require consideration. The study highlighted patient requests for more synchronous contact and to increase contact frequency. Further research is needed to evaluate the impact of support modality and frequency on the effectiveness and cost-effectiveness of ICBT for EDs. While asynchronous communication suits many patients, those requiring synchronous interactions may benefit from alternative formats. One potential advancement is expanding ICBT-E into Blended CBT-E, which integrates digital and synchronous components. This approach offers a promising solution by balancing ICBT-E's flexibility with enhanced support (Erbe et al., 2017). Stepped-care models integrating ICBT-E offer another effective and cost-efficient alternative, optimizing resource allocation while addressing varying patient needs (Pehlivan et al., 2022).

## Declaration of Generative AI and AI-assisted technologies in the writing process

During the preparation of this work the authors used ChatGPT in the writing process to improve the readability and language of the manuscript. After using this tool/service, the authors reviewed and edited the content as needed and take(s) full responsibility for the content of the published article.

## Declaration of competing interest

The authors declare that they have no known competing financial interests or personal relationships that could have appeared to influence the work reported in this paper.

## Appendix A Supplementary data

Supplementary material Image 1
