# Supplementary material for: Patient experiences of internet-based enhanced cognitive behavior therapy for eating disorders
Source: Internet Interv. 2025 Jan 23;39:100801. doi: 10.1016/j.invent.2025.100801 (PMC11795102; doi:10.1016/j.invent.2025.100801)
Supplement: Supplementary file 1 — Supplementary material [file mmc1.docx]

**INTERVIEW GUIDE**

1. What was your overall experience with the internet treatment ICBT-E?
2. What did you expect from the treatment before it started?
3. Did the treatment meet your expectations, or did you feel that something was missing?
4. Describe any changes you made during the course of the treatment.
5. Which parts of the treatment do you think contributed to these changes?
6. Which parts of the content in ICBT-E did you find helpful?
7. Which parts of the content in ICBT-E did you find less helpful?
8. Is there anything you think could be done to improve the less helpful parts? If so, what?
9. While you were undergoing the treatment, what made you continue/not continue with it?
10. How did you perceive the support from your therapist?
11. What did your therapist do that you found helpful?
12. What did your therapist do that you found unhelpful?
13. What could your therapist have done differently to make you feel more supported?
14. Did you experience any negative events or effects that you believe were related to the ICBT-E treatment?
15. If you experienced negative events or effects, could you describe them and specify when during the treatment they occurred, how often they occurred, and how long they lasted?
16. What was your attitude towards CBT for eating disorders (CBT-E) before the treatment?
17. What is your attitude towards CBT-E now after the treatment, and how has it changed as a result of the treatment?
18. What are your thoughts on undergoing treatment via the internet?
